# Puerperal Fever

**Published:** 1880-02

**Authors:** 


Puerperal Fever.—Dr. A. Green, of St. Louis, in *St Louis Medical and Surgical Journal*, January 5, has an article on this affection. After discussing the etiology, which is usually infection conveyed by the accoucheur and brought in contact with an abraded surface and the prophylaxis, which is mainly an evoidence of this manner of infection, he gives his treatment, which consists of vaginal injections of carbolic acid and water, two grs. to the ounce and washing the vulva and vicinity with the same, every two or three hours, administration of quinia, grs. xxv to xl in twenty-four hours, keeping the bowels open, and cold or hot applications to the abdomen, according to comfort. The following is the concluding portion of the report. "I here give the summary of Dr. L. C. Fritsch's remarks which I would recommend to every reader:

1. By every puerperal woman, the vagina ought to be kept clean from the lochia post partum.

2. After intra-uterine operations, by rotten children, by badly smelling lochia and when the fever has already begun, the uterus must be cleaned out also.

3. In spite of the most severe symptoms of septicæmia, there is yet hope for cure.

The doctor answers the objections raised against intra-uterine injections, with the exception of one, which I think is a very grave one, namely the danger of the fluid passing through the fallopian tubes into the peritoneal cavity. I think that we ought to make use of a dangerous remedy only when the danger from the disease is surely greater than that which may arise from the remedy. Allow me to analyze the second part of the summary of the doctor's remarks.

*a.* Intra-uterine operations. I endorse this only when these operations have been severe ones.

*b.* Rotten children: Only when they have remained in the uterus so long after the liquor amnii has escaped and air admitted that decomposition has taken place before delivery was accomplished.

*c.* Badly smelling lochia: Only when the intra-vaginal injections do not remove the smell, and

*d.* When the fever has already begun: I would use intra-uterine irrigations only when the puerperal fever commences soon after delivery and there is reason to think that the infection took place in the uterus itself; but when the fever commences on the third or fourth day after delivery, it is more than probable that the infection took place post partum, through the examining finger of the physician or midwife, either at the entrance of the vagina or in some little rents of the mucous membrane of the vagina itself, or at some point in the cervix where the examining finger can reach; and those parts that can be reached by the finger we can also reach by intra-vaginal injection.—*Toledo Medical and Surgical Journal.*

